# Subsequent Vaccination against SARS-CoV-2 after Vaccine-Induced Immune Thrombotic Thrombocytopenia

**DOI:** 10.3390/jcm13185462

**Published:** 2024-09-14

**Authors:** Günalp Uzun, Theresa Ringelmann, Stefanie Hammer, Jan Zlamal, Beate Luz, Marc E. Wolf, Hans Henkes, Tamam Bakchoul, Karina Althaus

**Affiliations:** 1 Centre for Clinical Transfusion Medicine Tuebingen, 72076 Tuebingen, Germany; 2 Institute for Clinical and Experimental Transfusion Medicine, Medical Faculty of Tuebingen, University Hospital of Tuebingen, 72076 Tuebingen, Germany; 3 Institute of Transfusion Medicine, Klinikum Stuttgart, 70174 Stuttgart, Germany; 4 Department of Neurology, Klinikum Stuttgart, 70174 Stuttgart, Germany; 5 Department of Neuroradiology, Klinikum Stuttgart, 70174 Stuttgart, Germany

**Keywords:** platelet factor 4, COVID-19 vaccines, SARS-CoV-2, thrombocytopenia, thrombosis

## Abstract

**Background:** Vaccine-induced immune thrombotic thrombocytopenia (VITT) is a rare but severe complication following vaccination with adenovirus vector-based COVID-19 vaccines. Antibodies directed against platelet factor 4 (PF4) are thought to be responsible for platelet activation and subsequent thromboembolic events in these patients. Since a single vaccination does not lead to sufficient immunization, subsequent vaccinations against COVID-19 have been recommended. However, concerns exist regarding the possible development of a new thromboembolic episode after subsequent vaccinations in VITT patients. **Methods:** We prospectively analyzed follow-up data from four VITT patients (three women and one man; median age, 44 years [range, 22 to 62 years]) who subsequently received additional COVID-19 vaccines. Platelet counts, anti-PF4/heparin antibody level measurements, and a functional platelet activation assay were performed at each follow-up visit. Additionally, we conducted a literature review and summarized similar reports on the outcome of subsequent vaccinations in patients with VITT. **Results:** The patients had developed thrombocytopenia and thrombosis 4 to 17 days after the first vaccination with ChAdOx1 nCoV-19. The optical densities (ODs) of anti-PF4/heparin antibodies decreased with time, and three out of four patients tested negative within 4 months. One patient remained positive even after 10 months post first vaccination. All four patients received an mRNA-based vaccine as a second vaccination against SARS-CoV-2. No significant drop in platelet count or new thromboembolic complications were observed during follow-up. We identified seven publications reporting subsequent COVID-19 vaccination in VITT patients. None of the patients developed thrombocytopenia or thrombosis after the subsequent vaccination. **Conclusion:** Subsequent vaccination with an mRNA vaccine appears to be safe in VITT patients.

## 1. Introduction

The emergence of Coronavirus Disease 2019 (COVID-19), an infectious disease caused by the Severe Acute Respiratory Syndrome Coronavirus 2 (SARS-CoV-2) in Wuhan, China, in December 2019, marked a significant turning point in global health. The virus spread rapidly across the world, and on 11 March 2020, the World Health Organization (WHO) officially declared the COVID-19 outbreak a pandemic. During the COVID-19 pandemic, the urge for preventing new infections and severe cases of COVID-19 was high. Therefore, new vaccines were developed with unprecedented speed, and from the end of 2020 onwards four vaccines against SARS-CoV-2 were approved by the Food and Drug Administration (FDA) and the European Medicines Agency (EMA). They consisted of two mRNA-based vaccines, BNT162b2 (Pfizer–BioNTech) and mRNA-1273 (Moderna), and two recombinant adenoviral vaccines, ChAdOx1 nCoV-19 (AstraZeneca) and Ad26.COV2.S (Johnson & Johnson/Janssen). The initial randomized controlled trials leading to Emergency Use Authorization by the FDA in the USA [1] and authorization by the EMA in Europe [2] provided evidence for the safety of these vaccines, with no reported increase in the risk of thrombotic complications [3,4,5].

ChAdOx1 nCoV-19 was one of the first vaccines to be approved for use against COVID-19. It also gained prominence as the first vaccine to be widely available in large quantities for global vaccination campaigns. Within a short timeframe of just a few months, millions of doses were administered worldwide, with over 82 million doses being delivered in the European Union alone. However, shortly after this large-scale rollout, there were reports of several cases of thrombocytopenia and thrombotic events, particularly concerning cases of cerebral venous sinus thrombosis (CVST), in people who had received the ChAdOx1 nCoV-19 vaccine [6]. Following investigations into these cases, a new medical condition known as vaccine-induced immune thrombotic thrombocytopenia (VITT) was defined [6,7,8]. VITT is a rare but serious complication that can occur after vaccination with certain adenovirus vector-based COVID-19 vaccines. It is characterized by the presence of thrombotic events alongside a low blood count. These events typically occur within a window of 4 to 28 days after vaccination. ChAdOx1 nCoV-19 has been associated with the highest reported incidence of VITT compared to other COVID-19 vaccines [9]. Several hypotheses have been suggested to explain the association between ChAdOx1 nCoV-19 and VITT [10,11]. However, the exact pathophysiological mechanisms remain under investigation [12].

The clinical and serological presentation of VITT bears a striking resemblance to heparin-induced thrombocytopenia (HIT), except for the absence of recent heparin exposure. Furthermore, the thrombotic events occurred in VITT patients at unusual locations, with an especially high incidence in the sinus veins and the portal, hepatic and splanchnic veins. Notably, patients presenting with CVST have a significantly higher mortality risk compared to VITT patients without CVST [10]. Similar to HIT, immunoglobulin G (IgG) antibodies targeting platelet factor 4 (PF4) have been demonstrated in serum samples from VITT patients [6,7,8]. Anti-PF4 antibodies make an immune complex with PF4, triggering platelet activation via the Fc gamma receptor IIA (FcγRIIa). Importantly, the detection of these anti-PF4 antibodies is considered one of the key diagnostic criteria established for VITT.

According to the recommendations of the International Society on Thrombosis and Haemostasis Scientific and Standardization Subcommittee on Platelet Immunology, the diagnostic process should include an IgG-ELISA detecting antibodies against PF4 and a functional platelet activation assay, e.g., the serotonin release assay (SRA), heparin-induced platelet activation assay (HIPA), or the modified SRA/HIPA [13]. Rapid assays, which are commonly used for HIT, are not suitable for the diagnosis of VITT [14].

Most cases develop symptoms within the first two weeks after vaccination, primarily related to thrombocytopenia (such as petechiae and hematoma) or the site of thrombosis [15]. CVST, the most common site of thrombosis, often presents with severe headaches as the initial symptom. Intracranial hemorrhage, a serious complication associated with increased mortality, occurs in nearly a third of patients with CVST [15]. Depending on the affected vessel, VITT patients may experience altered mental status, focal neurological deficits (e.g., weakness or paralysis on one side), abdominal pain (splanchnic vein thrombosis), dyspnea (difficulty breathing), chest pain (pulmonary artery embolism), or leg pain/swelling (deep vein thrombosis).

Currently, there is no standardized treatment for VITT. Due to the high prothrombotic risk, patients should receive anticoagulation therapy [16]. Initial reports recommended avoiding heparin due to the similarities between HIT and VITT, until HIT is ruled out as the cause of acute thrombocytopenia/thrombosis [6,17]. However, subsequent studies have demonstrated that anti-PF4 antibodies in HIT and VITT bind different epitopes on PF4, and heparin actually reduces the binding of anti-PF4 antibodies in VITT [18,19]. The recent ISTH guideline on anticoagulation in COVID-19 indicates that for patients with VITT, using unfractionated heparin (UFH) or low-molecular-weight heparin (LMWH) is a reasonable option for reducing the risk of adverse outcomes when non-heparin anticoagulants are not available [14].

Supportive care to manage coagulopathy is crucial to prevent rapid clinical deterioration. Intravenous immunoglobulins (IVIGs) have shown efficacy in treating VITT by inhibiting platelet activation mediated by anti-PF4 antibodies. Additionally, plasma exchange has been reported as a successful intervention in case reports, suggesting it may be a viable option in refractory cases [12]. Platelet transfusions should be avoided, as they impose a high risk of thrombosis [20]. Improvements in the early diagnostic process and treatment of VITT patients with CVST have successfully reduced the mortality rate from almost 50% in the beginning to 22% [21].

Several key questions remain regarding the long-term management of VITT patients following the acute phase. The crucial question is whether subsequent vaccinations in VITT patients are safe. Regulatory agencies recommend a second dose of COVID-19 vaccines for full protection, as studies show it increases protection against symptomatic disease [22,23]. The effectiveness of vaccines against breakthrough infections reduces with time after vaccination [24], which led to the recommendation of booster vaccinations especially in older people and immunocompromised patients. It is important to determine if patients with VITT have a higher risk of thrombocytopenia or thrombosis after receiving a second or booster vaccine against COVID-19.

Since VITT is a rare condition, even small case studies can contribute significantly to our understanding of long-term disease course. This report details the clinical and serological follow-up of four patients who developed VITT after ChAdOx1 nCoV-19 vaccination and subsequently received BNT162b2 as a second dose. Additionally, we conducted a literature review and summarized similar reports on the outcome of second vaccinations in patients with VITT.

## 2. Materials and Methods

### 2.1. Patients

We prospectively followed 4 patients who developed VITT after the first immunization with ChAdOx1 nCoV-19 (Vaxzevria; AstraZeneca, London, UK). The diagnosis of VITT was serologically confirmed according to the recommendations of the International Society on Thrombosis and Haemostasis Scientific and Standardization Subcommittee on Platelet Immunology [13]. The level of anti–PF4/heparin antibodies was measured using an immunoglobulin G (IgG)-enzyme immune assay (EIA). The ability of the sera to activate platelets was tested using a modified heparin-induced platelet aggregation assay as described below [7]. The initial clinical presentation of Case #1 and Case #2 has been presented previously [25].

### 2.2. Anti-PF4/Heparin Enzyme-Linked Immunosorbent Assay (ELISA)

We quantified IgG antibodies to PF4/heparin using a commercial EIA kit (Zymutest HIA IgG, Hyphen Biomed, Neuville-sur-Oise, France), following the manufacturer’s protocol. A volume of 200 μL of diluted patient sample (1:100) and 50 μL of platelet lysate containing PF4 was added to microtiter plate wells coated with protamine sulfate and unfractionated heparin. The plate was incubated at room temperature for one hour. After washing to remove unbound antibodies, 200 μL of horseradish peroxidase-conjugated polyclonal antibody was added to the wells, followed by another one-hour incubation at room temperature. Subsequent washing removed unbound immunoconjugate. Immediately after washing, 200 μL of tetramethylbenzidine substrate was added to the wells. To halt color development, 50 μL of 0.45 M sulfuric acid was added. Optical density (OD) was measured at 450 nm using a microplate reader. An OD of 0.5 or higher was considered positive per the manufacturer’s criteria.

### 2.3. PF4-Induced Platelet Activation Assay (PIPA)

We assessed the ability of sera to activate platelets using a modified HIPA, as previously described [7,26]. Briefly, serum samples were tested with washed platelets from four different healthy donors, both in the absence of heparin (buffer alone) and in the presence of various heparin concentrations or PF4. For the PF4 test, washed platelets were pre-incubated with 50 μg/mL PF4 (ChromaTec GmbH, Greifswald, Germany) for 10 min at room temperature. Then, 20 μL of patient serum and 75 μL of washed platelets (300 × 10^3^ platelets/μL) were added to microtiter wells. Final concentrations in the wells were 0.2 IU/mL heparin, 100 IU/mL heparin, or 10 μg/mL PF4. Reactions in microtiter wells containing spherical stir bars were stirred at approximately 500 revolutions per minute (rpm). Wells were examined for loss of turbidity at five-minute intervals. A serum was deemed reactive (positive) if a shift from turbidity to transparency occurred within 30 min in at least two platelet suspensions. The observation period was 45 min. All patient sera were heat-inactivated at 56 °C for 30 min before testing.

### 2.4. Literature Review

A comprehensive literature search was conducted on 1 April 2024, using PubMed to identify relevant studies on the outcome of second vaccinations in patients who developed VITT following the ChAdOx1 nCoV-19 vaccination. The search strategy included the following Medical Subject Headings (MeSH) terms and keywords: “vaccine-induced immune thrombotic thrombocytopenia” (OR “VITT”), “COVID-19 vaccine”, “thrombosis”, and “thrombocytopenia”. To broaden the search further, we also conducted manual searches by reviewing the reference lists of published case reports and case series identified through the PubMed search. If multiple publications were identified reporting clinical outcomes from the same cohort of VITT patients, we included only the one with the largest cohort in our analysis.

### 2.5. Ethical Considerations

The study was conducted following the principles outlined in the Declaration of Helsinki and with approval from the Institutional Review Board of the University of Tuebingen (Date of approval: 26 March 2021; Approval number: 236/2021BO1).

## 3. Results

### 3.1. Clinical Presentation and Serological Diagnosis

**Figure 1:**
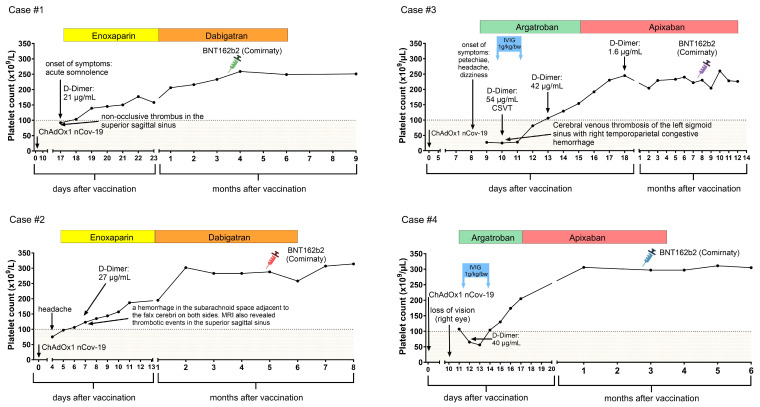
Platelet counts at initial presentation and during follow-up. All patients had a normal platelet count before the second vaccination, which was BNT162b2 (Pfizer-BioNTech) in all cases. No drop in platelet count or thrombosis was observed after subsequent SARS-CoV-2 vaccinations.

Four patients diagnosed with VITT were prospectively followed. Demographic and clinical data of cases are shown in Table 1. There were three female patients and one male patient. The median age was 44.5 years with a range of 22 to 62 years. All patients received the ChAdOx1 nCoV-19 vaccine as their first COVID-19 immunization. None of the patients had a confirmed COVID-19 infection before first vaccination. VITT symptoms occurred with a median of 10 days post vaccination, ranging from 4 to 17 days. CVST was diagnosed in three patients. The fourth patient presented with retinal artery occlusion. All patients had low platelet counts and elevated D-dimer levels at initial presentation. Initial anticoagulation treatment choices included low-molecular-weight heparin for two patients and argatroban for the remaining patients. Two cases also received IVIG. Platelet counts at initial presentation and during follow-up are shown in Figure 1.

**Figure 2:**
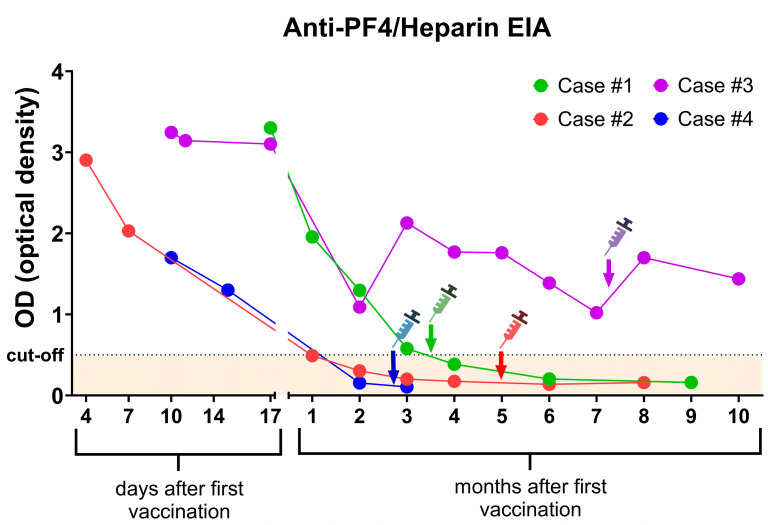
Time course of the PF4/heparin antibodies after diagnosis of VITT. The optical densities (ODs) of anti-PF4 antibodies decreased over time in subsequent follow-up examinations. Within 4 months, 3 out of 4 patients tested negative. One patient (Case #3) remained positive even 10 months after first vaccination. All patients received an mRNA vaccine for their second dose without experiencing new thromboembolic complications. The cut-off for anti-PF4/heparin EIA is 0.5 OD. The time point of the second vaccination is indicated with an arrow.

All patients demonstrated strongly positive anti-PF4/heparin antibody levels. Antibodies against platelet glycoproteins were not detected. Furthermore, a modified functional platelet activation assay (PIPA) was positive in all cases, confirming the clinical diagnosis of VITT. During follow-up, anti-PF4 antibody levels gradually decreased, falling below the cut-off level in three patients within 4 months after the first vaccination (Figure 2). One patient had detectable anti-PF4 antibodies at the last follow-up (10 months after the first vaccination). PIPA was negative after two months in Cases 1 and 4, and after three months in Case #3. In Case #2, PIPA was positive at the last follow-up after eight months.

### 3.2. Subsequent Vaccination

All patients participated in a shared decision-making process regarding revaccination for COVID-19. They were informed about the potential benefits, including developing an adequate antibody response for protection against severe COVID-19 and the possibility of VITT recurrence with thrombocytopenia as well as thrombosis. While we recommended revaccination with an mRNA-based COVID-19 vaccine, we strongly discouraged getting an adenoviral vector-based COVID-19 vaccine again. A pre-vaccination platelet count was obtained to rule out active thrombocytopenia and establish a baseline. The second vaccination was recommended to be administered while on ongoing anticoagulation therapy. Following vaccination, close platelet count monitoring was advised at the family physician or at the local hospital up to 4 weeks after vaccination. The characteristics of the study participants with regard to the first and second vaccination and serological testing are shown in Table 2.

All patients received an mRNA-based COVID-19 vaccine (BNT162b2) as the second vaccination (Table 2). Pre-vaccination platelet counts were within the normal range in all patients, and no post vaccination thrombocytopenia was observed. None of the patients developed a thrombotic complication. Anti-PF4/heparin antibody levels were below the cut-off value in three patients, and their OD values remained unchanged following the second vaccination. Case #3, however, maintained a detectable anti-PF4/heparin antibody, and a slight increase in OD was observed after revaccination. This patient did not experience a VITT relapse either.

### 3.3. Literature Review

Our literature review identified seven studies that investigated revaccination in patients diagnosed with VITT (Table 3). Together, these studies reported on 103 patients who underwent revaccination with COVID-19 after a diagnosis of VITT. A total of 132 vaccinations were administered in these patients. The median follow-up after revaccination varied widely between the studies, ranging from 10.5 weeks to 79 weeks.

Together with the current study, the majority of patients (120, 92.3%) initially received the ChAdOx1-S/nCoV-19 vaccine (AstraZeneca). A smaller cohort (8, 6.2%) received the Ad26.COV2.S (Johnson & Johnson) vaccine as their first dose. Information on the initial vaccine type was not available for two patients. For subsequent vaccinations, mRNA-based vaccines were predominantly used. The BNT162b2 vaccine (Pfizer-BioNTech) was administered in 112 cases (82.4%). Other revaccinations included the Moderna (Spikevax) vaccine (10, 7.4%) and unspecified mRNA vaccines (9, 6.6%). Of note, five patients (3.7%) received the ChAdOx1-S/nCoV-19 vaccine again.

Data on anti-PF4/heparin antibody levels before and after vaccination were sparse. In the current study and three others, these antibody levels in 98% of reported cases either decreased or remained stable after revaccination. A slight increase in the anti-PF4 OD was observed in Case #3 (Figure 2). This finding suggests a favorable immunological response to subsequent vaccine doses in VITT patients.

Complications following revaccination were minimal. Three patients experienced a mild decrease in platelet count (thrombocytopenia) after receiving subsequent vaccines, but no cases of thrombosis were reported [27]. This suggests a low risk of serious adverse events associated with revaccination in this patient population.

## 4. Discussion

This study reports the outcomes of second COVID-19 vaccinations in patients with VITT. None of our patients developed thrombocytopenia or thrombosis following mRNA-based COVID-19 vaccination. A literature review identified seven additional studies investigating revaccination in VITT patients. Collectively, these studies and our cohort encompass 107 patients without recurrent VITT after further COVID-19 vaccinations.

Numerous questions persist regarding the optimal management of VITT. Of particular interest is the role of subsequent COVID-19 vaccinations in patient outcomes. Both domestic and international health authorities recommend at least two COVID-19 vaccinations for complete immunization, with additional doses to maintain adequate antibody levels, especially among the elderly and immunocompromised. Supporting the importance of continued vaccination in VITT patients, a recent study reported a higher breakthrough infection rate among VITT patients who did not receive a second vaccine compared to those who subsequently received one or two mRNA vaccine doses (29% vs. 9%) [34].

The selection of the vaccine for subsequent immunization in VITT is crucial. All patients in our cohort received mRNA-based COVID-19 vaccines, and we recommend avoiding adenovirus vector-based vaccines. While most VITT cases occur after the first dose of ChAdOx1 nCoV-19, VITT following a second vaccination has also been documented. Lacy et al. reported five patients who received a second dose of ChAdOx1 nCoV-19 after a confirmed (n = 1) or possible (n = 4) VITT [28]. No relapse has been reported. In our literature review, other than these five patients reported by Lacy et al., all other VITT patients received mRNA-based vaccines (Table 3). Although a number of cases with VITT-like presentations following mRNA vaccination have been reported, a consensus among experts has not been reached. Our findings, combined with the literature, suggest the safety of mRNA vaccines as a subsequent vaccination option for patients with VITT.

The optimal timing of subsequent vaccination merits consideration. Whether to delay vaccination until anti-PF4 antibody levels decline below cut-off remains an open question. Anti-PF4 antibodies and functional assays, such as serotonin-release assay or PF4-induced platelet activation assay, are commonly employed as markers of disease activity. Previous studies indicate that anti-PF4 antibody levels typically decrease over time [27,35]. Functional platelet-activating antibodies exhibit a more rapid decline (median 15.5–16 weeks) compared to anti-PF4 antibodies detected by EIA (median 24 weeks) [27,35]. Nevertheless, a subset of patients exhibits persistently elevated antibody levels [36]. Similarly, in our cohort, the functional platelet activation assay was negative within 3 months in three cases, but it was positive after 8 months in one patient. Despite this, most patients can safely receive subsequent mRNA COVID-19 vaccinations without complications [31,35]. In our cohort, three patients had negative EIA at the time of vaccination. One patient with a positive EIA (Case #3) experienced an increase in anti-PF4 OD post vaccination without developing thrombocytopenia. The patient did not report any viral infections at follow-up. This increase could be due to post vaccination inflammation. Since no significant change in platelet count was reported, we believe that this increase in the antibody level did not have clinical relevance in Case #3. These findings suggest that vaccination may be feasible despite persistent antibodies, although individual risk assessment for thrombosis or bleeding is essential.

Given the hypercoagulable state associated with VITT, anticoagulation is standard care during the acute phase. However, the optimal duration of anticoagulation especially in the absence of thrombosis remains controversial, with reports of persistent and recurrent cases in the literature [27,37,38]. In our cohort, anticoagulation was maintained until anti-PF4 antibody levels declined, and all patients received subsequent vaccination while on anticoagulation. Similarly, the UK cohort [28] reported no instances of recurrent thrombocytopenia or thrombosis among patients vaccinated while receiving anticoagulant therapy.

The strengths of this study include its long-term follow-up, comprehensive assessment of anti-PF4 antibodies, and functional platelet activation testing, all of which contribute significantly to a deeper understanding of the long-term consequences of VITT and inform potential revaccination strategies. Additionally, the inclusion of a thorough literature review provides a broad perspective on the current understanding of VITT and revaccination outcomes. However, the relatively small sample size, a direct consequence of the rarity of VITT, limits final conclusions. Moreover, heterogeneity in reporting methods and follow-up durations across the studies introduces potential biases and affects the comparability of the results. Limited data on anti-PF4/heparin antibody levels and the lack of large-scale studies further constrain the robustness of the conclusions. These factors should be considered when interpreting the findings and developing future research directions.

## 5. Conclusions

Current evidence suggests that revaccination with mRNA-based COVID-19 vaccines in patients with a history of VITT is safe. However, meticulous monitoring of platelet counts and clinical signs of thrombosis after the vaccination is of utmost importance. Consultation with a coagulation disorders specialist is strongly recommended. Despite these promising findings, further research is essential to confirm these results and to develop optimal revaccination strategies for patients with a history of VITT.

## Figures and Tables

**Table 1:** Demographic and clinical data of cases with VITT.

Case	Age	Sex	Symptom Onset (Days)	Thrombosis	Platelet Count (150–450 × 10^9^/L)	Anti-PF4/Heparin EIA (OD)	PF4-HIPA
1	36	f	17	CVST	92	3.3	+
2	22	f	4	CVST	75	2.9	+
3	57	f	9	CVST	25	3.2	+
4	62	m	10	RAO, muscle vein thrombosis	56	1.7	+

CVST: cerebral venous sinus thrombosis; EIA: enzyme immune assay; HIPA: heparin-induced platelet activation assay; OD: optical density, RAO: retinal artery occlusion.

**Table 2:** Characteristics of study participants regarding the first and second vaccination as well as serological investigations.

Parameter	Case #1	Case #2	Case #3	Case #4
First vaccine	ChAdOx1 nCoV-19	ChAdOx1 nCoV-19	ChAdOx1 nCoV-19	ChAdOx1 nCoV-19
Treatment				
Anticoagulation	Initially Enoxaparin, then Dabigatran	Initially Enoxaparin, then Dabigatran	Initially Argatroban, then Apixaban	Initially Argatroban, then Apixaban
Intravenous immunoglobulin	-	-	+	+
Second vaccine	BNT162b2	BNT162b2	BNT162b2	BNT162b2
Time to second vaccination (weeks)	17	21	33	12
Anticoagulation at the time of second vaccination	+	+	+	+
Diagnostics				
Anti-PF4/heparin EIA (OD) before and after second vaccination				
Before	0.579	0.174	1.02	0.155
After	0.386	0.137	1.7	0.106
Platelet count before and after second vaccination (10^9^/L)				
Before	233	302	222	306
After	259	288	230	297
Follow-up after second vaccination				
Follow-up duration after second vaccination (weeks)	24	17	24	17
Thrombocytopenia	-	-	-	-
Thrombosis after second vaccination	-	-	-	-

**Table 3:** Summary of published case series reporting safety of revaccination following VITT.

Parameter		Schönborn et al. [27]	Lacy et al. [28]	Panagiota et al. [29]	Arachchillage et al. [30]	Lindhoff-Last et al. [31]	Lotti et al. [32]	Abou-Ismail et al. [33]	Current Study	Total
Patients receiving subsequent vaccination		48/71	40	5	4	3	2	1	4	107
Number of subsequent vaccinations		77	40	5	4	3	2	1	4	136
Median follow-up time after first vaccination(weeks)		79	n.a.	42.9	56	22.5	10.5	11	24	
Type of first vaccination	ChAdOx1-S/nCoV-19	64	40	5	4	3	n.a.	0	4	120
	Ad26.COV2.S	7	0	0	0	0	n.a.	1	0	8
Subsequent vaccination	BioNTech/Comirnaty	67	33	0	4	3	0	1	4	112
	Moderna/Spikevax	8	2	0	0	0	0	0	0	10
	mRNA (not specified)	2	0	5	0	0	2	0	0	9
	ChAdOx1-S/nCoV-19	0	5	0	0	0	0	0	0	5
Anti-PF4/heparin EIA reported	before	n.a.	n.a.	n.a.	4	3	n.a.	1	4	12
	after	decreasing	n.a.	n.a.	4	3	n.a.	1	4	12
Complications after subsequent vaccination	Thrombocytopenia	3 (minor reduction)	n.a.	n.a.	0	0	n.a.	0	0	3
	Thrombosis	0	0	0	0	0	0	0	0	0

PF4: Platelet factor 4; VITT: vaccine-induced immune thrombotic thrombocytopenia; n.a.: not available.

## Data Availability

Data may be requested for academic collaboration from the corresponding author.
